# Early Vasopressin Application in Shock study

**DOI:** 10.1186/cc13348

**Published:** 2014-03-17

**Authors:** S Oliveira, F Dessa, C Rocha, F Oliveira

**Affiliations:** 1 Hospital Bangu, Rio de Janeiro, Brazil

## Introduction

Vasopressin is frequently used to maintain blood pressure in refractory septic shock [1]. This study is looking into the hypothesis that vasopressin compared with norepinephrine would decrease the severity of septic status, evolution to multiple organ dysfunction, length of hospitalization, and mortality among patients with septic shock.

## Methods

In this randomized, double-blind study, we assigned patients who still needed vasopressor to restore tissue perfusion after fluid resuscitation to receive norepinephrine (0.05 to 2.0 μg/kg/minute) or vasopressin (0.01 to 0.03 U/minute) with low doses of norepinephrine. Both groups had the vasoactive drug infusions titrated and tapered to maintain a target mean blood pressure. The analysis included the total time of use and dosage of vasopressors every 6 hours, the move to single organ dysfunction and multiple organ failure, length of ICU stay and hospitalization, and mortality 7, 14 and 28 days after the start of infusions.

## Results

A total of 407 patients underwent randomization but 387 patients were included in this study (191 patients received vasopressin, and 196 received norepinephrine only). The total time for vasopressors was 37 hours and 68 hours in the vasopressin and norepinephrine groups with *P *= 0.02. Single organ dysfunction and multiple organ dysfunction using vasopressin and norepinephrine were respectively: 37.7% vs. 49.2%, *P *= 0.02; and 17.8% vs. 26%; *P *= 0.05. Length of stay in the ICU was 14 and 17 days (*P *= 0.29) and the time of hospitalization was 23 and 28 days (*P *= 0.11) respectively. There was a significant difference between the vasopressin and norepinephrine groups in the mortality rate at 14 and 28 days (29.3% vs. 36.7%, *P *= 0.05; 34% and 42.3%, *P *= 0.03), but there were no significant differences in the overall rates for 7-day mortality (21.2% vs. 23.9%, respectively; *P *= 1.1).

## Conclusion

Early application of vasopressin reduced the time of vasopressor use, progression to multiple organ dysfunction, length of stay in the ICU, and mortality rates at 14 and 28 days as compared with norepinephrine only. This observed difference can be attributed to early restoration of tissue perfusion in the control group making the state of septic shock shorter and reducing the potential for multiple organ dysfunction, which directly influenced patient survival.

## References

[B1] Dellinger RP Levy MM Carlet JM Surviving Sepsis Campaign: International guidelines for management of severe sepsis and septic shock Crit Care Med 2008 36 296 327 10.1097/01.CCM.0000298158.12101.41 18158437

